# Monitoring resilience in bursts

**DOI:** 10.1073/pnas.2407148121

**Published:** 2024-07-24

**Authors:** Clara Delecroix, Egbert H. van Nes, Marten Scheffer, Ingrid A. van de Leemput

**Affiliations:** ^a^Department of Environmental Sciences, Wageningen University and Research, Wageningen 6700 AA, The Netherlands

**Keywords:** resilience, DIORs, tipping points, monitoring, resource allocation

## Abstract

The possibility to anticipate critical transitions through detecting loss of resilience has attracted attention in many fields. Resilience indicators rely on the mathematical concept of critical slowing down, which means that a system recovers more slowly from external perturbations when it gets closer to tipping point. This decrease in recovery rate can be reflected in rising autocorrelation and variance in data. To test whether resilience is changing, resilience indicators are often calculated using a moving window in long, continuous time series of the system. However, for some systems, it may be more feasible to collect several high-resolution time series in short periods of time, i.e., in bursts. Resilience indicators can then be calculated to detect a change of resilience between such bursts. Here, we compare the performance of both methods using simulated data and showcase the possible use of bursts in a case study using mood data to anticipate depression in a patient. With the same number of data points, the burst approach outperformed the moving window method, suggesting that it is possible to downsample the continuous time series and still signal an upcoming transition. We suggest guidelines to design an optimal sampling strategy. Our results imply that using bursts of data instead of continuous time series may improve the capacity to detect changes in resilience. This method is promising for a variety of fields, such as human health, epidemiology, or ecology, where continuous monitoring can be costly or unfeasible.

## Significance

Gauging the risk of tipping points is of relevance in complex systems ranging from health to climate. For this purpose, dynamical indicators of resilience are being derived from long continuous time series to monitor the system and obtain early warning signals. However, gathering such data can be prohibitively expensive or practically unfeasible. Here, we show that collecting data in brief, intense bursts may often solve the problem, making it possible to estimate change in resilience between the bursts with relatively high precision. This may be useful, for instance, in psychology, where brief bursts of mood could help anticipate future depressive episodes; and also in epidemiology where short bursts of active case surveillance may be used to anticipate disease outbreaks.

Many complex systems such as ecosystems, financial markets, and human health, can show sudden changes punctuating seemingly stable times. Some of these abrupt shifts are critical transitions where the system moves between two alternative stable states. Such changes can be induced by a gradual change in an underlying condition, bringing the system closer to a critical threshold (1). A range of complex systems have been suggested to undergo such critical transitions. Examples include the start and end of depression episodes (2, 3), extinction of populations in ecosystems (4), and disease outbreaks (5).

Systems that gradually approach a critical threshold will progressively lose resilience. In other words, the maximum magnitude of a perturbation that the system can stand without tipping to an alternative attractor becomes smaller (6, 7). This maximum magnitude of a perturbation is also referred to as the width of the basin of attraction of a particular state. Mathematically, it has been shown that a decrease in the width of the basin of attraction results in a decrease in the speed of the system close to the equilibrium, a phenomenon called critical slowing down (7, 8). This can be intuitively understood when imagining the speed of a ball rolling toward a valley in a deep, wide basin compared to a shallow, narrow basin. Thus, a decreasing recovery rate of a system pushed by external perturbations may indicate that the system is more likely to go through a critical transition (8, 9). As it is often impossible to measure the recovery rate directly (e.g., by perturbing the system experimentally), statistical metrics that capture changes in the fluctuations resulting from the natural regime of stochastic perturbations are used as a proxy. Autocorrelation and variance are the most used indicators of critical slowing down in such data. Both indicators are expected to increase when a system gradually approaches a critical transition (10, 11).

Typically, to calculate trends in resilience indicators, long time series are analyzed using a moving window approach (11). In other words, indicators are calculated repeatedly in overlapping subsets of the data to follow their evolution over time. For such calculations, one should use a time series of a relevant variable describing the state of a system. For example, proxies of climate such as temperature or deuterium content have been used to anticipate transitions (12). Resilience indicators calculated from reconstructed climate time series based on these proxies showed a significant trend before abrupt shifts, such as the end of glaciation periods (12). Changes in resilience indicators have, for instance, also been detected in incidence time series prior to the reemergence of malaria in Kericho, Kenya (13), and in mood time series prior to the start of depressive episodes (2, 3).

Although resilience indicators are a valuable model-free anticipation method, they are not suitable for all situations. Various situations exist where critical slowing down cannot be expected prior to an abrupt shift (10). Most importantly, resilience indicators cannot be expected to serve as a warning signal if there is no gradual change in an underlying condition. Even if a gradual change in resilience exists, long high-resolution equidistant time series with small observation errors are required for the method to work well. The number of data points should be high enough to observe a change in the system (11). Additionally, for measuring autocorrelation, the resolution of the time series should be appropriate to observe the time scale of the system’s dynamics (10). As monitoring is generally costly, such data requirements are rarely met for some systems. When patients are involved, collecting long, uninterrupted time series may also be challenging. For instance, only in exceptional cases a time series of the mood of a patient is long enough and has a high enough resolution to detect a change in resilience indicators prior to depression episodes (3). Obtaining the required length and resolution of a time series of mood is very demanding and will lead to high dropout rates.

Here, we explore the alternative to use bursts of data instead of continuous time series to monitor a potential loss of resilience in a system. We define bursts as short periods of intense monitoring in a system, leading to multiple short time series of high resolution. We estimate resilience within each of the bursts using autocorrelation and variance and subsequently compare bursts. We discuss when monitoring in bursts can be more efficient, and how results depend on the number and duration of the bursts, their resolution, and the interval between two bursts. As the costs per burst and sample vary and can influence the way to allocate sampling resources, we investigate the effect of various scenarios on the performance of the burst approach for monitoring resilience. Monitoring resilience in bursts could, for instance, help to overcome challenges in monitoring disease transmission with imperfect data from passive surveillance, being replaced with more accurate burst data of active surveillance at similar costs (5). Another example is that measuring in bursts could help to reduce dropout rates when monitoring the resilience of patients (14).

We used a well-studied model with a tipping point to generate high-resolution time series. From those time series, we extract subsamples to systematically compare different approaches for monitoring resilience. For a complementary empirical example, we apply the burst approach to a long time series of self-reported moods used to predict the onset of a depressive episode (3). Finally, we formulate advice on how to monitor resilience depending on the system setup and available monitoring resources.

## Materials and Methods

### Synthetic Data.

To compare the performance of resilience indicators with both the rolling window and burst approach, we first generated long-term high-resolution time series using a commonly used model with critical transitions, referred to as the overgrazing model.

We used a simple one-variable model described by May et al. (15). The model is a generic stochastic differential equation model capturing the growth dynamics of vegetation under grazing pressure (Eq. **1**).

[1] $$\frac{\mathrm{dN}}{\mathrm{dt}}=rN\left(1-\frac{N}{K}\right)-c\frac{N^{2}}{H^{2}+N^{2}}+\sigma \frac{\mathrm{dW}}{\mathrm{dt}}.$$

The vegetation *N* grows logistically, and the grazing rate follows a sigmoidal saturation curve. At low vegetation density, grazing is limited by vegetation availability, while at high vegetation density, grazing is limited by the digestion rate of herbivores. In other words, the grazing rate increases with vegetation density until it reaches a maximum. *N* represents the density of vegetation, parameter *r* the maximum growth rate, *K* the carrying capacity of the population growth, *c* the maximum grazing rate, and *H* the grazing saturation constant.

Stochasticity is included in the model to reproduce natural fluctuations as additive noise. We used a Wiener process (dW/dt) with a fixed SD σ. This model was implemented in Grind for Matlab (16).

For the default parameter settings used in this paper, the deterministic version of the model (σ = 0) displays two-fold bifurcations (F1 and F2 in *SI Appendix*, Fig. S1). The high-density vegetation state undergoes a critical transition when the maximum grazing rate c crosses the critical threshold F1.

To simulate a loss of resilience of the high vegetation state, we generated long time series with c gradually increasing toward its critical threshold, from 0.5 to 2.2. Other parameter values are fixed on r = 1, K = 10, H = 1, and σ = 0.5. The model was simulated for 8,000 d with three data points per day, resulting in time series with 24,000 data points. A number of 100 stochastic realizations of the time series were simulated to quantify the performance over 100 repetitions. As a control, we generated time series with a fixed c = 0.5, representing a system with steady resilience.

After simulation, we simulated measurement error by adding a random number from a standard normal distribution (mean 0 and SD σ_ME_ 0.5) to each data point in the generated time series.

### Subsampling the Master Time Series.

We see the generated high-resolution time series as representing the actual dynamics of a system and use the resulting ‘master’ time series to sample 1) bursts for the burst approach and 2) continuous time series for the rolling window approach by using subsets of the data.

To make a fair comparison between approaches, we tried to keep the total number of samples the same for each method (Table 1). When comparing the burst approach with the rolling window approach, this implies that in the rolling window approach, either the master time series is truncated (scenario I) or the data resolution is lower (scenario II). The former leads to a truncated control parameter range (Δ_c_) over the time series. To be complete, we also did the rolling window analysis for the whole master time series (scenario III), although this implies that we use more samples in this analysis. Thus, for each scenario, two of the three sampling choices (Δ_1_, Δ_c_, and L) were the same for both approaches and one differed (Table 1).

**Table 1.** Summary of values for the number of data points (L), the data resolution (Δ_1_), and the difference in resilience (Δ_c_) for the burst approach and the three scenarios of the rolling window approach

	Δ_1_ (sampling interval)	Δ_c_ (gradient of c values)	L (total number of data points)
Burst approach	1 d	1.7	1,000
Rolling window approach Scenario I, truncating	1 d	0.21	1,000
Rolling window approach Scenario II, thinning	24 d	1.7	1,000
Rolling window approach Scenario III, gold standard	1 d	1.7	8,000

**Fig. 1.**
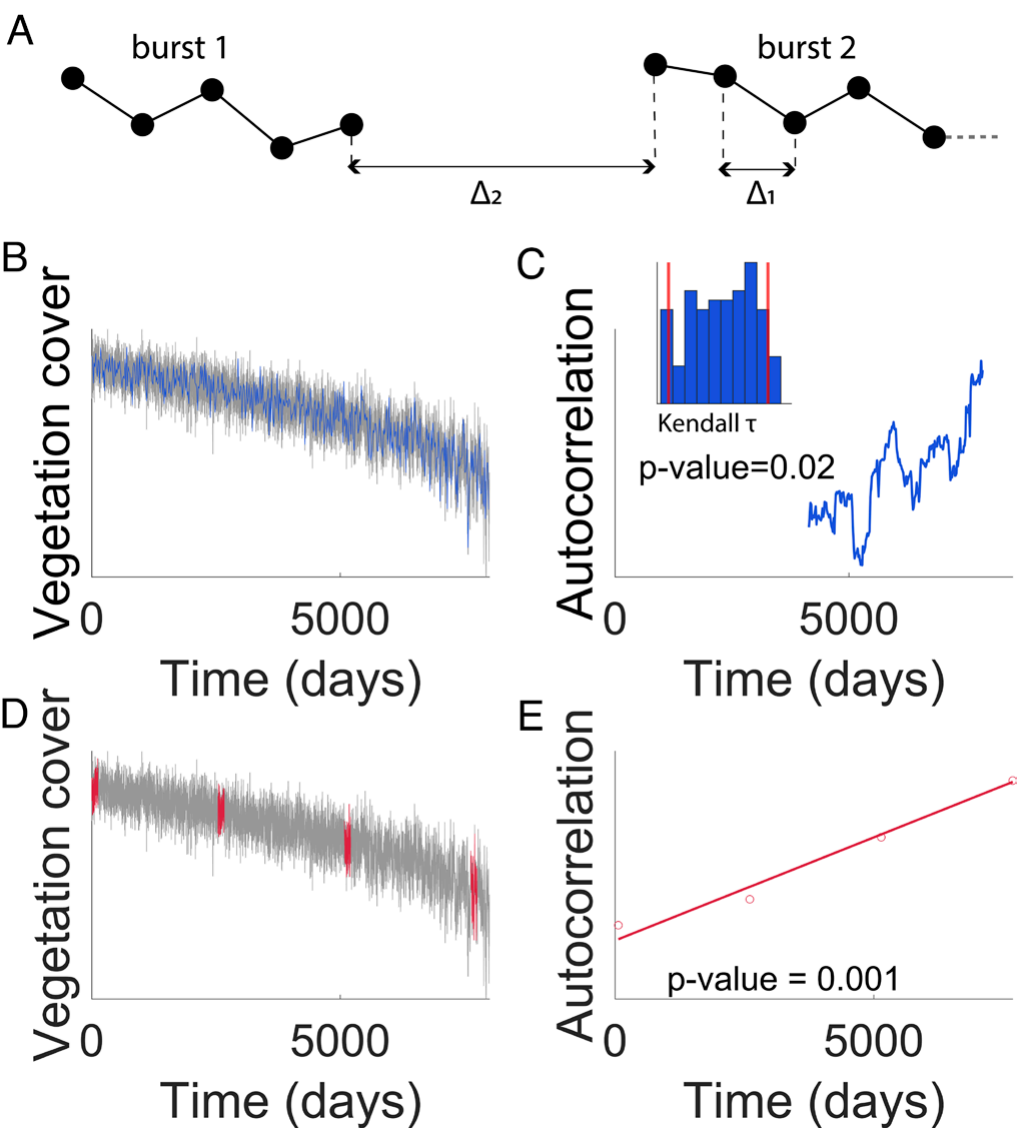
Summary of the types of monitoring to anticipate a transition, with associated analysis methods. (*A*) Graphical representation of monitoring parameters of interest, Δ_1_ and Δ_2_. The circles symbolize the collected samples. (*B*) Example of samples collected continuously for the rolling window approach. Master time series in gray and sampled time series in blue. (*C*) Measure of the autocorrelation using a rolling window for the dataset represented in *B*. The histogram represents the distribution of Kendall tau values in the datasets of the null model. (*D*) Example of samples collected in four bursts for the burst approach. Master time series in gray and sampled bursts in red. (*E*) Graphical representation of the method used to detect a loss of resilience between several bursts for the dataset represented in *D*. For each burst, we estimate the indicator. Then, we estimate the slope of the regression for the set of points. We also calculate the *P*-value of the slope being different from zero.

We also investigated the effect of several sampling choices on the performance of the burst method (Fig. 1): the interval between two samples in a burst Δ_1_ (further referred to as sampling interval) and the interval between two bursts Δ_2_, which is proportional to the difference in resilience (i.e., because we assume an underlying linear loss of resilience over time), quantified as the difference in bifurcation parameter between the bursts Δ_c_. Additionally, we varied the number of bursts *n*, the number of samples in a burst *λ*, and the total number of samples collected *L* = *n λ*.

### Empirical Data.

In addition to the simulation study, we used a real dataset to compare the proposed burst approach to the conventional method (3, 17). In this study, a patient subject to depressive episodes monitored his daily life experiences up to 10 times a day at a random moment for 239 d through a 50-item questionnaire. On average, the interval between two data points was 3 h and 40 min, and 6.2 data points were collected daily. The study comprised five phases: a baseline period of 28 d, a double-blind period with no reduction of the antidepressant intake, a period during which the patient’s antidepressant intake was progressively reduced in double-blind, a postassessment period, and a follow-up period. The monitoring times were randomly chosen daily, thus, the data points are not equidistant. Additionally, the patient’s depressive symptoms were measured on Mondays, and a significant shift in depressive symptoms was detected on day 127 of the experiments. At the end, 829 data points are available before the shift.

In this study, we focused on the items pertaining to mood states, self-esteem, and physical condition. More specifically, we focused on negative feelings (feeling down, irritated, lonely, anxious, suspicious, guilty, doubtful, restless, agitated, worried, ashamed, doubting myself, tired, in pain, dizzy, having a dry mouth, and nauseous) as previous work suggests that changing fluctuations in those symptoms may best signal an upcoming depressive episode (2). These affects were all correlated positively with one another. We discarded the items dizzy, dry mouth, and nauseous as no variation was shown in these affects. With the remaining 14 items, we performed a PCA as a data reduction technique to obtain one time series of 829 data points before the shift. The PCA coefficients were calculated on the baseline period only (the first 28 d, 177 data points), and the time series were projected in the direction of the first principal component. Similarly to the simulation study, we subsampled from the master time series to compare the burst and the rolling window approach. We used the previously defined downsampling scenarios I, II, and III for the rolling window approach.

### Resilience Indicators.

To measure resilience, we used indicators of critical slowing down (9, 11, 18). We specifically considered the performance of variance and autocorrelation as resilience indicators and performed significance tests using different methods for continuous monitoring and burst monitoring (see below).

#### *Significance test for continuous monitoring*: *Rolling window approach*.

For detrended continuous time series, we calculated the autocorrelation and variance with a rolling window in the classical way, using an implementation in Matlab (https://git.wur.nl/sparcs/generic_ews-for-matlab). We used a window size of 50% the size of the time series as this is the most common in literature. The trend in the indicators was evaluated using the Kendall tau value. To assess the significance of the tau values calculated in nonindependent data, we used null models to generate surrogate datasets The null hypothesis that we test is that the conditions are stationary while having a similar power spectrum as the original dataset. Therefore, the surrogate datasets are created using a Fourier transform of the whole dataset, by keeping the same power spectrum as the original dataset but randomizing the phases. The *P*-value is then calculated as the proportion of surrogate datasets leading to a stronger trend (quantified using a Kendall tau correlation coefficient) in the resilience than in the original dataset. This method has been described and used extensively in previous studies (11, 19). A trend was considered significant if its *P*-value was below the 5% threshold.

#### *Significance test to compare bursts of data*: *Burst approach*.

If bursts of data were collected, we used the slope of a Theil-Sen regression of the indicators with the rank of each burst to assess a loss of resilience over time. Specifically, we calculated the indicators variance and autocorrelation for each burst and fitted a Theil Sen regression over the indicators of all the bursts. The slope of the regression allows to quantify a potential increase of the indicator over all the bursts. The Theil Sen regression is insensitive to outliers and is more robust than traditional regressions for skewed data. The slope is calculated as the median of the slopes of all pairs of points (20).

We used a sieve bootstrapping algorithm to assess the significance of the decrease in resilience in the burst approach, preserving the autocorrelation structure of the time series. The bootstrapping algorithm consists of two steps (21). After detrending the data, an AR(n) model is fitted, and the data residuals are determined. Second, the AR(n) model is used to generate the boostrapped time series, where stochasticity is included by drawing residuals of the AR(n) model with replacement. We generated bootstrapped time series for each burst and used these time series to calculate the value of the indicator. Then, we fitted a Theil-Sen regression over the indicators of the bootstrapped bursts and obtained the *P*-value of the probability of the slope being higher than zero. A loss of resilience was considered significant if its *P*-value was below the 5% threshold.

#### *Measure of the performance*.

To quantify the performance of both approaches, the analyses were repeated for 100 generated time series to estimate the true positive rate (sensitivity) and true negative rate (specificity). For the true positive rate, we used time series with an increasing c, representing a loss of resilience. We calculated the true positive rate as the proportion of repetitions resulting in a *P*-value below 0.05. For the true negative rate, we used time series with a fixed c = 0.5, representing stable time series. We defined the true negative rate as the proportion of repetitions resulting in a *P*-value above 0.05.

## Results

### Performance of the Burst Method vs. the Rolling Window Method.

**Fig. 2.**
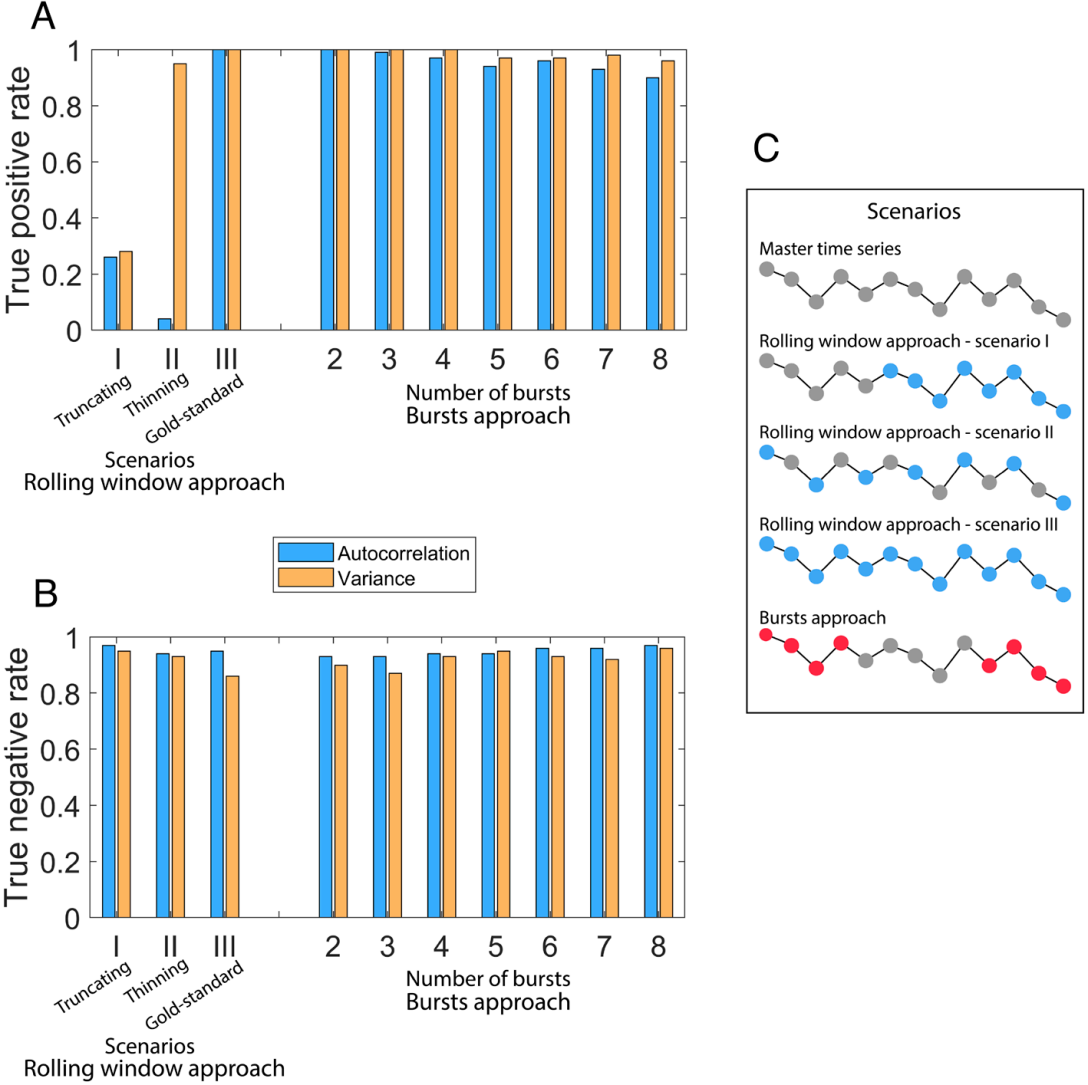
Performance of resilience indicators (autocorrelation and variance) depending on the sampling approach. For the rolling window approach, we use three scenarios for comparison (Table 1): scenario I, truncating, has the same total number of data points and resolution as the burst approach but covers a shorter gradient (i.e., only the last part of the master time series), scenario II, thinning, has the same number of data points and covers the full gradient from high to low resilience but a different resolution, and “gold-standard” scenario III has the same resolution and gradient but a higher number of data points. For the burst approach, we vary the number of bursts. Except in scenario III of the rolling window approach, the total number of data points is L = 1,000 for both approaches, regardless of the number of bursts. Except in scenario II of the rolling window approach, the data points are subsampled with a daily resolution (Δ1 = 1 d). For the burst approach, the data points are equally allocated between the bursts, and the bursts are equally spaced in the time series. (*A*) True positive rate. The true positive rate is calculated in data simulated using the model [Eq. (**1**), parameter Table 1], with c increasing from 0.5 (high resilience) to 2.2 (low resilience) over 100 repetitions. (*B*) True negative rate. The true negative rate is calculated in simulated data with a fixed c = 0.5 over 100 repetitions. (*C*) Illustration of the allocation of data points for the rolling window approach in scenarios I, II, and III and for the burst approach by subsampling from the master time series. Gray points are points in the master time series that are unused in that approach. Colored points are used.

In our simulations, the burst approach signals an upcoming transition as often as our gold standard scenario (I) for the rolling window approach (Fig. 2*A*) and produces false alarms as rarely as the rolling window approach (Fig. 2*B*). Increasing the number of bursts reduces the true positive rate of variance (the true positive rate decreased by 8% between two bursts and eight bursts) and autocorrelation (the true positive rate decreased by 12% between two bursts and eight bursts) but does not seem to affect the true negative rate significantly. To compare the burst approach with the rolling window approach, we used three sampling scenarios for the rolling window approach (*Materials and Methods*, Table 1, and Fig. 2*C*). Truncating the time series (scenario I) hinders the performance of the rolling window approach for both indicators. By contrast, reducing the resolution (scenario II) mainly affects the performance of autocorrelation for the rolling window approach. Therefore, bursts can enhance the performance of autocorrelation as a higher resolution is reached with the same total number of samples. Not surprisingly, in the gold-standard scenario III, where neither effort nor time is limiting, the rolling window approach can signal upcoming transitions best, with a true positive rate of 100%.

These analyses were repeated in a dataset generated using the vegetation model including additional complexities: i) using a nonlinear increase of the bifurcation parameter, ii) with autocorrelated noise, and iii) subject to periodic forcing (*SI Appendix*, Figs. S3–S6). The first two complexities decreased the overall performance of both the rolling window approach and the burst approach, as these constitute violations of the basic conditions necessary to apply resilience indicators. Adding periodic forcing did not affect results of the gold standard scenario from the moving window approach but did hinder the performance of the burst approach especially when bursts were subsampled without taking the period of the fluctuations into account (*SI Appendix*, Fig. S6).

Additionally, we repeated the analyses in a dataset generated using the FAMOUS model, a complex model simulating the Atlantic meridional overturning circulation (AMOC) (22). In this dataset, the AMOC is subject to a “hosing experiment,” gradually increasing the freshwater influx until the circulation collapses (23). The rolling window approach using the whole dataset (scenario III, gold-standard) exhibited only weak signals. Similarly, downsampling the data to use the burst approach and the rolling window approach with scenarios II and III exhibited weak signals prior to the collapse (*SI Appendix*, Fig. S7). This exercise illustrates that the utility of the burst approach is not limited to simple idealized models but also that signs of critical slowing down which are hardly detectable in a full dataset should not be expected to appear more clearly if only part of the data is used. Details and full results of the AMOC analysis may be found in *SI Appendix*.

### An Empirical Example: Mood Fluctuations Before Depression.

We also tested the burst approach against a conventional rolling window approach using an existing dataset of mood changes (3). Using the rolling window approach on the full processed dataset, we found a significant rise in variance (*P*-value = 0.01) before the transition into depression, consistent with previous findings (3). Autocorrelation showed a weak signal when computed on the raw data, ignoring nonequidistant spacing (*P*-value = 0.056). However, significance increased when we used the daily mean value (*P*-value = 0.03, see *SI Appendix*), possibly due to the fact that this corrected for daily cycles and made data intervals equidistant. As aggregating per day left too little data to perform the burst analysis, we proceeded with variance to compare the bursts vs the moving window approach.

**Fig. 3.**
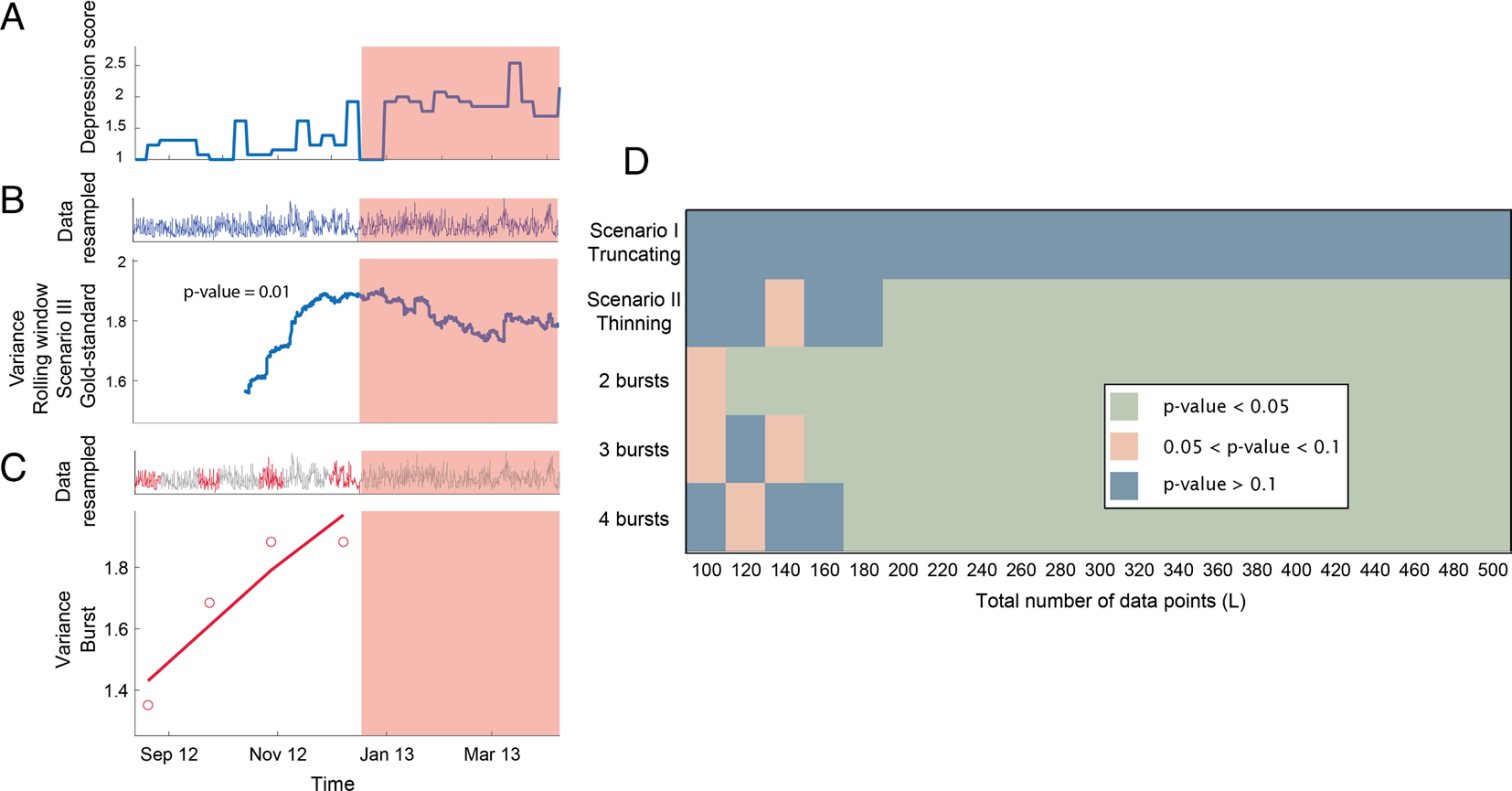
Analysis of the empirical dataset, mood fluctuations of a patient measured for 239 d through a 50-item questionnaire. (*A*) Depression score over time; the shaded region indicates the tipping point. (*B*) Data used for the rolling window approach, scenario I, and trend of variance. The shaded region indicates the tipping point, 829 data points are available before the tipping point. (*C*) Data used for the burst approach and value of variance for each burst as well as the linear regression. (*D*) Comparison of the rolling window approach and burst approach in anticipating a depressive episode using mood data from Wichers and Groot (3) when subsampling from the master time series under scenario I: truncating, and II: thinning (rolling window approach) and for different number of bursts (burst approach). The performance of the approach is quantified using the p-value of the Ebisuzaki test (19) for the rolling window approach and of the slope being significantly different than zero for the burst approach. Scenarios for the rolling window approach are summarized in Table 1 and depicted in Fig. 2*C*.

The results illustrate that consistent with the findings from simulated data, the burst approach could signal an upcoming transition (Fig. 3). When subsampling the original 829 data points, the burst approach could detect the transition down to 120 data points for two bursts, 160 for three, and 180 for four bursts. Thus, our real data example illustrates that, indeed, in this field where continuous monitoring can be too demanding, the burst approach can be a good alternative. Details on our methods and full results are presented in see *SI Appendix*.

### Effect of the Sampling Interval.

**Fig. 4.**
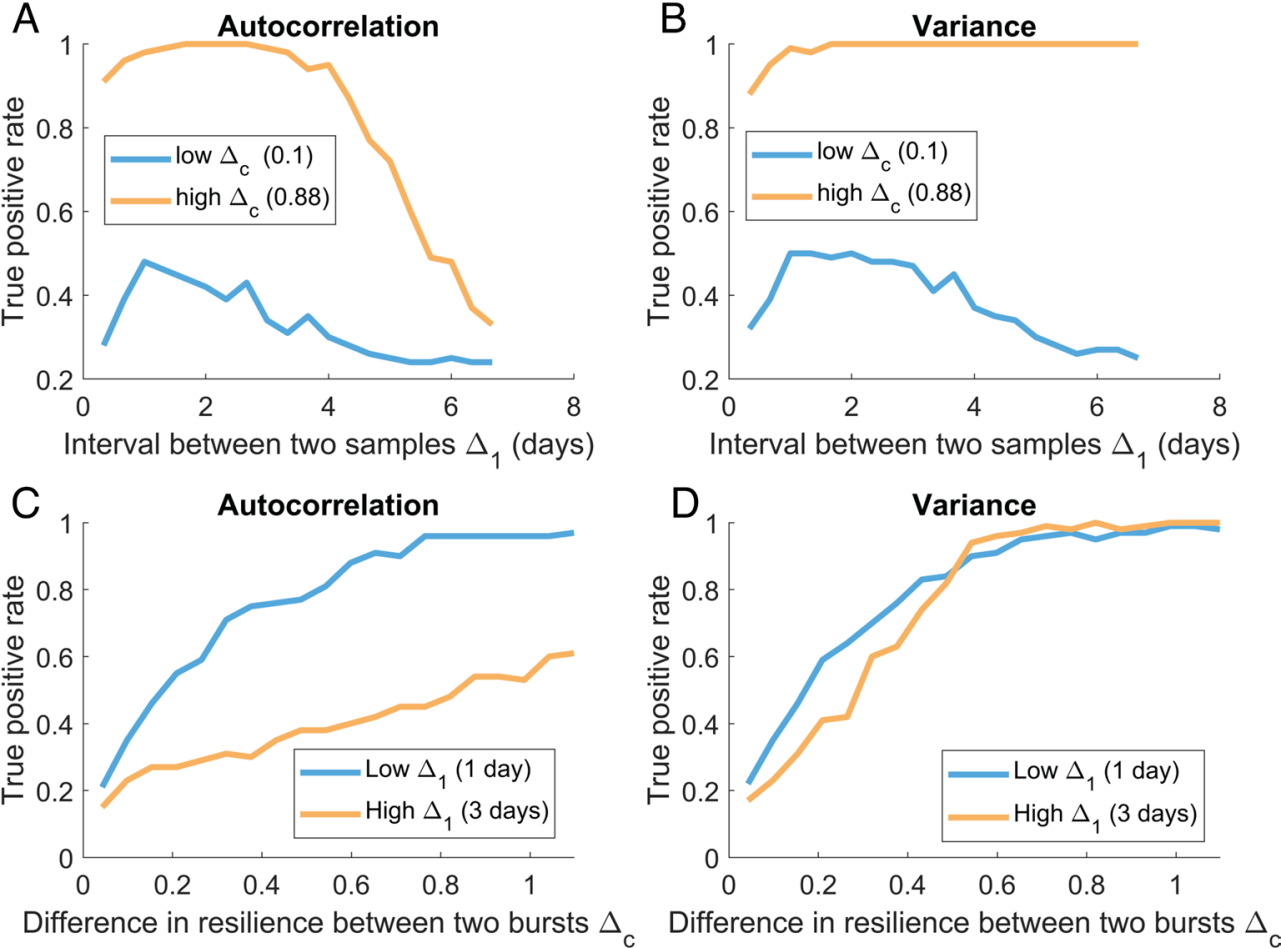
Effect of the sampling interval Δ_1_ (*A* and *B*) and the difference in resilience between two bursts Δ_c_ (*C* and *D*) on the performance of the burst approach. The performance is quantified using the true positive rate over 100 repetitions, using subsamples from the master time series. The total number of data points was constant (L = 1,000) and spread equally between two bursts. (*A*) Performance of autocorrelation depending on the interval between two samples Δ_1_. We estimated that performance for two values of Δ_c_. (*B*) Performance of variance depending on the interval between two samples Δ_1_. We estimated that performance for two values of Δ_c_. (*C*) Performance of autocorrelation depending on the difference in resilience between two bursts Δ_c_. We estimated that performance for two values of Δ_1_. (*D*) Performance of variance depending on the difference in resilience between two bursts Δ_c_. We estimated that performance for two values of Δ_1_.

We sampled from the simulated master time series to investigate the sampling interval (i.e., the interval between two samples within a burst) on the performance of the burst approach (Fig. 4 *A* and *B*). Our results suggest that the sampling interval only affects the autocorrelation. To optimize the performance of autocorrelation, there is an optimal sampling interval. This is consistent with previous results (11) and additional analyses provided in supplementary (*SI Appendix*, Fig. S2). Variance was hardly affected by the sampling interval.

### Effect of the Interval Between Bursts.

Additionally, we investigated the effect of the interval between two bursts on the performance of the burst approach (Fig. 4 *C* and *D*). In our model setup, a longer interval between two bursts implies a larger difference in resilience between the two bursts, which improved the detection of a loss of resilience for both variance and autocorrelation. There was no effect of the interaction of the sampling interval and the interval between two bursts, as the same patterns were observed for the different combinations of conditions.

### Effect of the Number of Bursts.

To explore trade-offs in sampling strategies, we analyzed different combinations of choices under cost restraints.

#### *Fixed total number of samples L*.

**Fig. 5.**
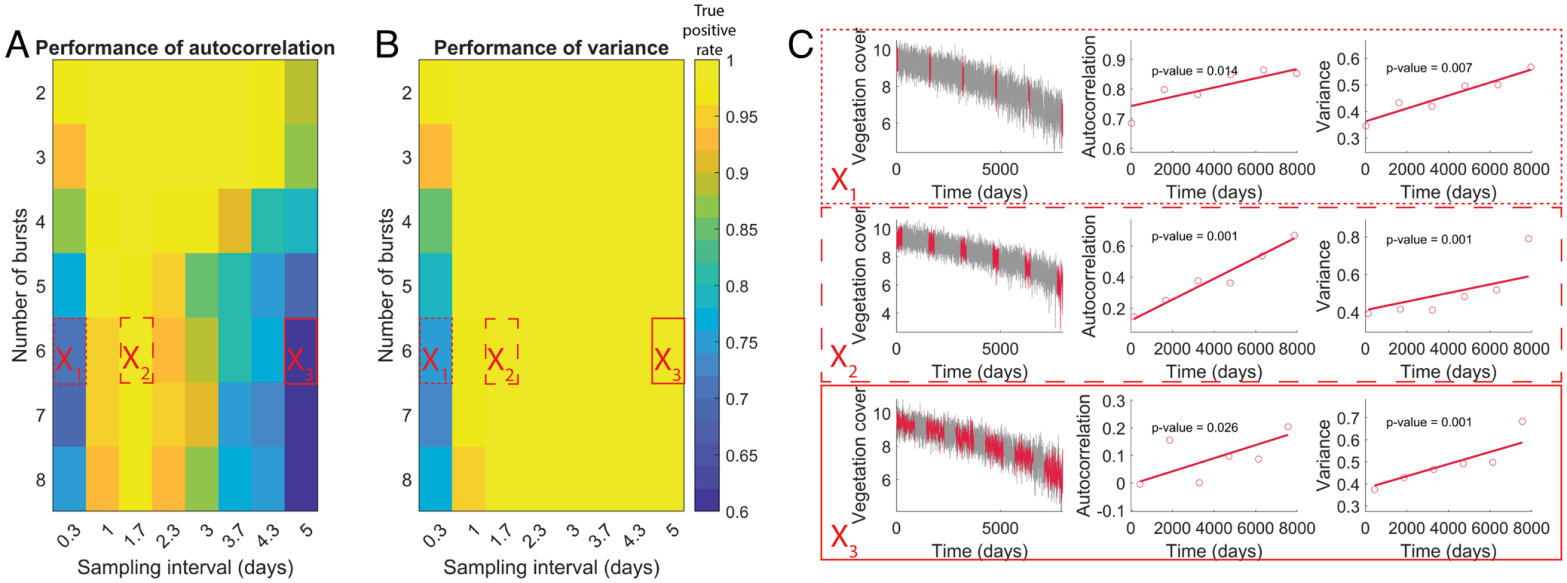
True positive rate of the burst approach depending on the number of bursts n and sampling interval Δ_1_. The true positive rate is estimated using the time series generated with the vegetation model over 100 repetitions. The total number of data points was constant (L = 1,000), regardless of the number of bursts. The bursts are equally spaced in time. (*A*) Performance of the autocorrelation. (*B*) Performance of the variance. (*C*) Example of subsampling in the master time series for three combinations of parameters specified in *A* and *B*.

First, we investigated the case where the total number of samples *L* is fixed (Fig. 5), as could be the case when each collected sample has a high cost. Under this constraint, a too-high number of bursts hampers the detection of a loss of resilience, especially for autocorrelation, as the bursts become too short and do not have enough data points to be representative of the state of the system. However, a lower number of bursts results in a later detection of the critical transition (*SI Appendix*, Fig. S12). Additionally, consistent with previous results (Fig. 4 *A* and *B*), the performance of autocorrelation diminishes for a long sampling interval. These results suggest that there is an optimal way of allocating *L* samples when monitoring resilience in bursts, with high enough resolution and large enough bursts with a big enough interval between each burst. As more bursts increase the lead time of prediction but decrease the prediction performance, a compromise has to be found depending on the objectives of the study. Similar results were observed for different values of the total amount of data points *L* (*SI Appendix*, Fig. S10), where, not surprisingly, a decrease in the overall true positive rate was observed for a decrease in *L*.

#### *Fixed number of samples per burst λ*.

**Fig. 6.**
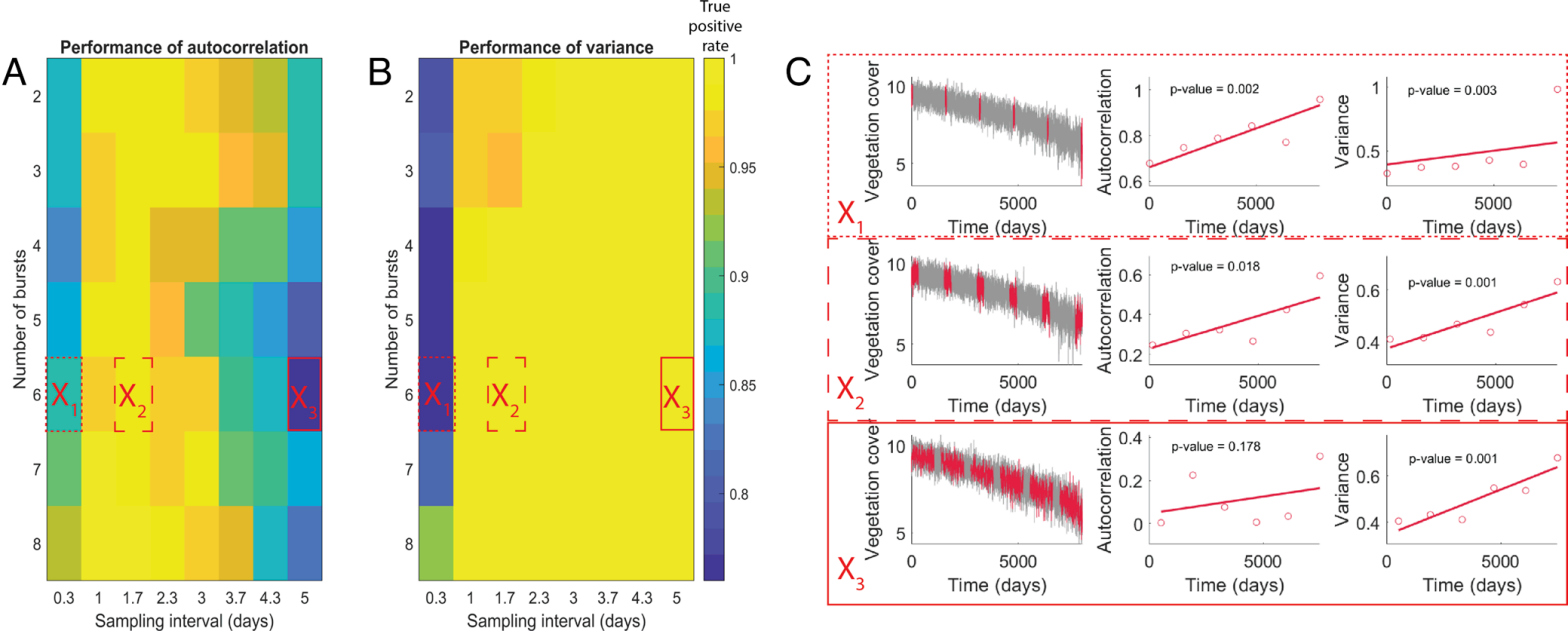
True positive rate of the burst approach depending on the number of bursts n and sampling interval Δ_1_. The true positive rate is estimated using the time series generated with the vegetation model over 100 repetitions. The number of data points per burst was constant (λ = 200), regardless of the number of bursts. The bursts are equally spaced in time. (*A*) Performance of the autocorrelation. (*B*) Performance of the variance. (*C*) Example of subsampling in the master time series for three combinations of parameters specified in *A* and *B*.

In addition, we investigated a scenario where costs are purely determined by the number of bursts, keeping the number of samples per burst constant (Fig. 6). This scenario is relevant when an expensive device must be rented for the monitoring of a burst or a patient should come to the hospital for each burst. Opposed to the previous scenario, the number of bursts did not affect the performance (as it did not affect the number of samples per burst), but still affected the lead time of prediction (*SI Appendix*, Fig. S13). Under our assumption of a linear loss of resilience, large numbers of bursts coupled with large sampling intervals led to a shorter interval between the bursts and, thus, a smaller loss of resilience between each burst, hampering the signal. Similar results were observed for different values of the amount of data points per burst *λ* (*SI Appendix*, Fig. S11), yet a decrease in the overall true positive rate was observed for a decrease in *λ*.

## Discussion

Our results imply that using bursts (i.e., two or more short high-resolution time series) to monitor resilience can be a useful alternative to the more common sampling of long, continuous time series analyzed using a rolling window approach (Fig. 1). This result was found for both simulated (Fig. 2) and real-life data (Fig. 3). Since reaching the data quality standards required for the rolling window approach can be prohibitive (10, 24), the apparent power of the burst approach may expand opportunities to monitor resilience (Table 2). In most situations, the burst approach will not beat results from full-flung permanent monitoring. However, under some trade-offs the burst approach may enhance detection power. For instance, given a limited total number of data points, concentrating the efforts in bursts allows increasing the resolution of collected time series. High resolution can be essential to detect changes in autocorrelation (Fig. 4), which is a more direct and robust indicator of resilience than variance (25).

**Table 2.** Example of applications for bursts and moving indicators of resilience

	Objective	Monitoring in bursts	Continuous monitoring	Reference
Vegetation	Anticipate a decline in vegetation due to overharvesting	Bursts of monitoring the fluctuations in vegetation cover over shorter periods of time (or spatial snapshots)	Continuous monitoring of the amount of vegetation	26
Depression	Anticipate episodes of depression using mood-tracking	Investigate a potential loss of resilience by using snapshots of mood fluctuations for a patient, reducing the dropout rate	Use continuous, long-term mood tracking and investigate the trend in resilience indicators	2, 3
Epidemiology	Anticipate epidemics using incidence time series	Compare incidence time series with previous years or use bursts of active surveillance	Anticipate an outbreak in a specific area using long-term data	5
Resilience of human body: aging	Anticipate a decline in the elderly using data from balance boards or grip strength, for instance	Investigate a loss of resilience in a patient using bursts of data collected regularly by placing the patient on a balance board	In that case, it is impossible to monitor continuously the balance or grip strength of a patient	27, 28
Resilience of human body: chronic diseases	Anticipate acute episodes of chronic diseases using biomarker data over time	Collect bursts of biomarker concentration in a patient to investigate a loss of resilience	Estimate biomarker concentration regularly over a long time period (may cause inconvenience for patients and higher risk of dropouts)	27, 29
Resilience of human body: hypertension	Anticipate upcoming hypertensive crisis using blood pressure time series	Collect bursts of blood pressure in a patient	Estimate blood pressure regularly over a long time period (may cause inconvenience for patients and higher risk of dropouts)	27, 30
Resilience of human body: sports	Anticipate athletes’ loss of resilience using physiological and psychological data	Collect bursts of physiological and psychological data to reduce the risk of dropout	Monitor physiological and psychological variables continuously over long time periods	14
Animal (livestock) resilience	Anticipate loss of resilience in livestock animals to increase animal welfare, using physiological variables	Collect bursts of physiological variables in livestock animals to reduce monitoring efforts by going to the farms less regularly for intense monitoring	Collect data regularly over long time periods to monitor a linear loss of resilience in the long run	27, 31

Obviously, fundamental limitations of resilience indicators also hold for the burst approach (10, 24, 32). Resilience indicators can only detect a change in the system caused by an underlying parameter gradually moving toward a critical transition. If the change is too fast or caused by noise, resilience indicators are of no use to detect that change (10).

Bursts may be especially useful for monitoring resilience of systems that are hard to measure continuously, such as patients. Monitoring resilience in human (mental) health can help anticipate critical transitions resulting in chronic diseases, depressive episodes, or loss of resilience due to unhealthy aging (27, 28). We refer here to resilience as a dynamic property of complex systems (27), which differs slightly from the definition generally used in psychopathology to refer to the human capacity to recover from traumatic events (33).

Our depression example illustrates that an episode could have been anticipated with much less burden to the subject than collecting the entire time series. This exceptional patient monitored their daily life experiences up to ten times a day for 239 d in a row (Fig. 3). Our analysis suggests that, for instance, two bursts of 12 d each spaced by 2 mo and using the resolution provided in the dataset (up to 10 times a day) would also have shown a significant warning prior to the start of the depressive episode (*SI Appendix*, Table S1). However, this was a controlled experiment, and a linear loss of resilience was expected as the patient gradually changed the intake of antidepressants. For patients who do not change their medication, a gradual loss of resilience before a depressive episode cannot always be expected, and thus not measured. Additionally, the dataset was analyzed retrospectively, knowing the moment of transition and subsampling bursts accordingly. In real life, since the moment of transition is not known, the timing of the bursts is crucial to be able to anticipate a transition and bad timing can lead to missed warnings. However, properly collected bursts of data with the right timing can reduce the monitoring burden for patients while still being able to anticipate upcoming critical transitions.

It is easy to think of other fields in which a burst approach might help to detect changes in resilience that could precede critical transitions. For instance, in epidemiology, resilience indicators have been shown to signal upcoming disease outbreaks, but data requirements are hardly ever met, especially in periods of low transmission when the disease is not being monitored (34). Actively seeking cases of a given disease in a population (active surveillance), provides better data to calculate resilience indicators, but this is extremely costly and therefore not feasible to do permanently. Bursts of active surveillance could allow to monitor the risk of disease outbreaks.

Bursts may also be useful to compare the resilience of different instances of a system, rather than the same system at different moments. For instance, short time series were used to show that autocorrelation and variance in postural balance time can discriminate between healthy and unhealthy aging in the elderly (28). Although they compared time series from different individuals, their approach is comparable to our burst approach, where shorter time series are collected to compare resilience in a system over time. Similarly, time series from satellite data were used to compare the resilience of tropical forests at many locations across the globe (35).

**Fig. 7.**
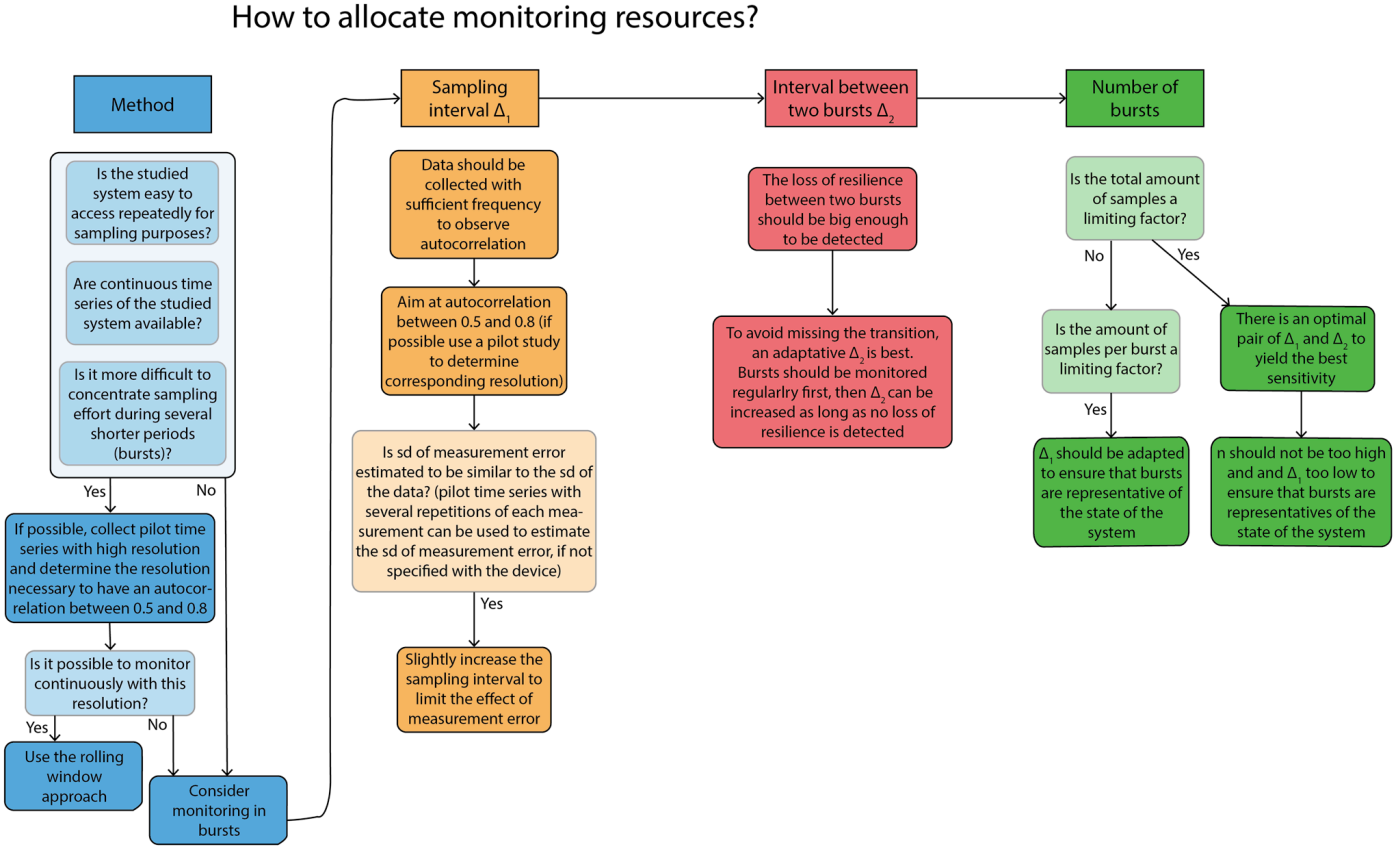
Decision tree to allocate sampling resources when monitoring a potential loss of resilience, when sampling resources are limiting.

While the burst approach can be useful, it is important to consider trade-offs between the number of bursts, length of burst, sampling interval, and interval between two bursts (Fig. 7). Optimal strategies depend on whether the costs per sample (Fig. 5) or the costs per burst (Fig. 6) are limiting. In the first case, lowering the number of bursts helps to make sure that there are enough data points in each of the bursts. When conditions change gradually, a larger interval between two bursts, results in a larger difference in resilience making it easier to detect a difference between the bursts (Fig. 4 *C* and *D*). However, obviously, a large interval between subsequent bursts also increases the risk of being too late for detecting the transition, as information about the resilience is not updated between the bursts. To help overcome that challenge, one could determine the interval between the bursts adaptively: starting with bursts close to one another and then increasing the interval between two bursts if there was little change in resilience visible. However, this only works for a gradual loss of resilience but can lead to a missed transition for an initially stable system suddenly losing resilience, as specified in the analyses of the empirical dataset.

In summary, monitoring in bursts can be a solution to the large data requirements of resilience indicators. The main limitation of the burst approach lies in the monitoring choices it requires, which can greatly affect the performance. Also, the lack of permanent monitoring implies that sudden changes of resilience may be missed. However, repeated bursts often outperform continuous monitoring of resilience when there are costs or practical limitations to data collection.

## Supplementary Material

Appendix 01 (PDF)

## Data Availability

Previously published data were used for this work (17).
