# Supplementary material for: Monitoring resilience in bursts
Source: Proc Natl Acad Sci U S A. 2024 Jul 24;121(31):e2407148121. doi: 10.1073/pnas.2407148121 (PMC11295040; doi:10.1073/pnas.2407148121)
Supplement: Supplementary file 1 — Appendix 01 (PDF) [file pnas.2407148121.sapp.pdf]

PNAS

Supporting Information for
Monitoring Resilience in Bursts

Clara Delecroix[1]*, Egbert H. van Nes[1], Marten Scheffer[1], Ingrid A van de Leemput[1]

*Clara Delecroix
Email: clara.delecroix@wur.nl

This PDF file includes:

Supporting text
Figures S1 to S13
Tables S1

Stability analysis of the model

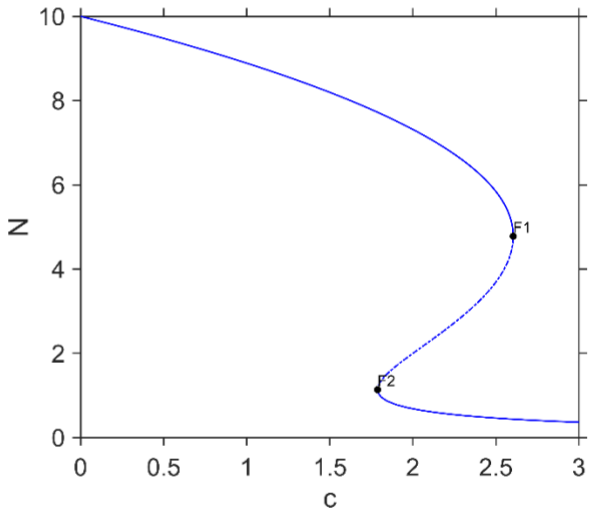

Fig. S1. Bifurcation of the deterministic version of the model with varying parameter c. This model has one stable high vegetation state for low levels of c, two stable states for intermediate values of c, and a low vegetation state for high c. At F1 (c=2.6) and F2 (c=1.8), the system crosses a fold bifurcation.

Effect of measurement error on the autocorrelation

Autoregressive model

We used an autoregressive model to investigate the effect of the sampling interval Δ_1 and the amplitude of the measurement error σ_{ME} on the accuracy of the estimation of autocorrelation.

$$n(t+1) = \alpha(n(t) - n_0) + n_0 + \sigma\varepsilon$$

(2)

In the model, α is the autocorrelation with lag 1, namely the correlation between two time points delayed with a lag of one, n0 represents the initial condition and $\sigma\varepsilon$ is white noise to reproduce intrinsic stochasticity. In this model, each datapoint depends on the previous datapoint. This model is a direct representation of the autocorrelation and helped us studying the effect of other factors on the autocorrelation straightforwardly. After simulating the time series, we added measurement error by sampling from a standard normal distribution for each time step, multiplied by an amplitude factor σ_{ME}. We used α=0.99, n0=900 and $\sigma\varepsilon = 1$, and several values for σ_{ME}=0, 1, 3, 5.

The effect of the sampling interval Δ_1 and the amplitude of measurement error σ_{ME} on was then investigated (Fig S1). Without measurement error, the estimation of autocorrelation is more accurate for a short sampling interval. However, including measurement error resulted in an optimal sampling interval to measure the autocorrelation with the best accuracy.

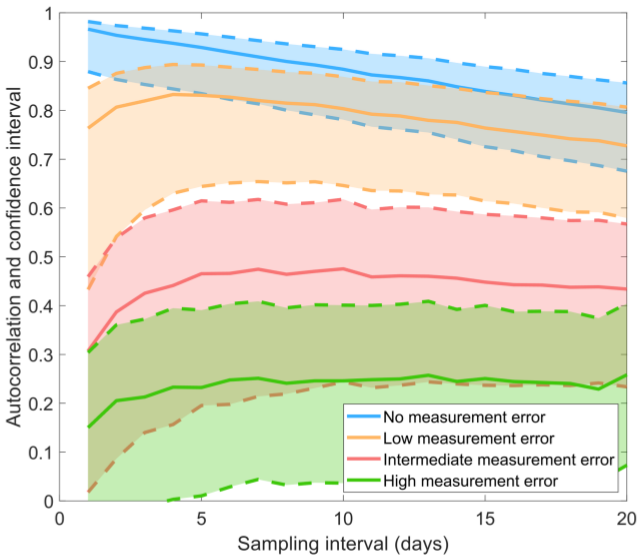

Fig. S2. Autocorrelation and its confidence interval depending on the sampling interval and measurement error, calculated in time series generated with the autoregressive model

Additional complexities in the vegetation model

To investigate the limitations of the burst approach, we include additional complexities to the vegetation model presented in the main text.

Different increase functions for the bifurcation parameter
In the main text, we assumed a linear increase of the bifurcation parameter *c* leading to a critical transition. This is one of the main requirement when using resilience indicators to anticipate a transition, however, we try to challenge that assumption to investigate how the indicators measured in bursts of data react.

- Saturating increase

We investigate a loss of resilience following a saturating curve over time. We use a Monod function defined as:

$$c(t) = c_{min} + c_{max}\frac{t}{t + K_s}$$

With c_{min}=0.5 the minimum value of *c*, c_{max}=2.2 the maximum value of *c* and K_s=0.1 the half saturation constant, meaning the time where the *c* reaches half of its maximum value. We show the evolution of *c* over time in Figure S3A.
A Monod increase of the bifurcation parameter leads to a decrease of the overall performance, for both the autocorrelation and variance, and both the rolling window approach and the burst approach. This is consistent with previous studies stating that resilience indicators can be used to detect a loss of resilience only when this loss is gradual over time (Dakos et al., 2015). This decrease in the overall performance is especially visible for the truncating scenario (decrease of 88% for autocorrelation and 71% for variance), but also for the burst approach (decrease of 6% for autocorrelation and 3% for variance compared to a linear loss of resilience, for the average over all numbers of bursts).

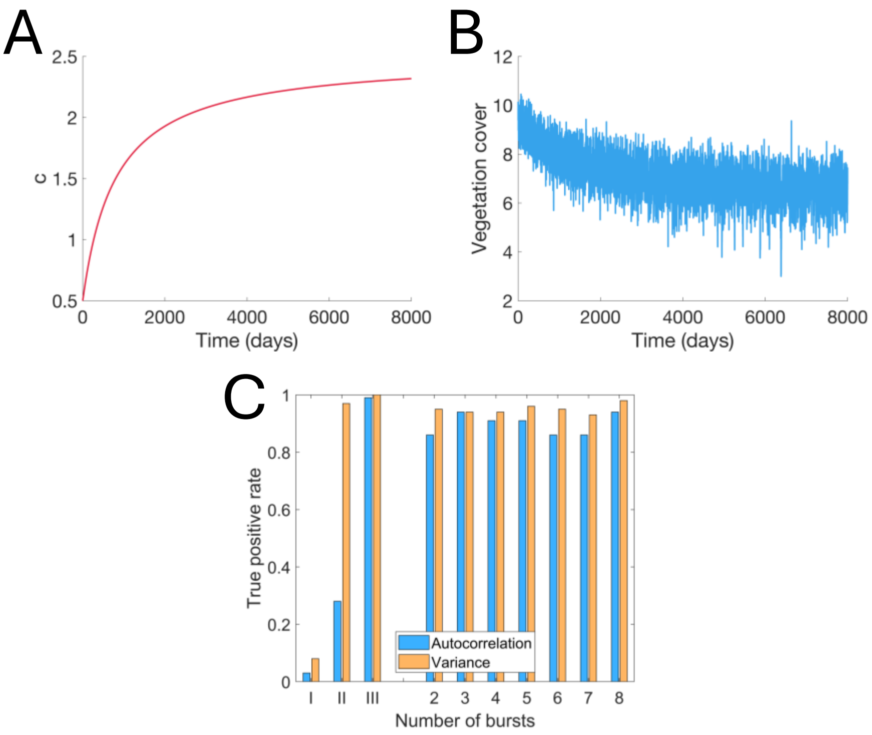

Fig. S3. Example of time series with a Monod increase of the bifurcation parameter, and corresponding performances for the rolling window approach and the burst approach. (A) Time series of the bifurcation parameter c, following a Monod function with a half velocity constant K_S=0.1t_{max}. (B) Time series of the vegetation cover. (C) True positive rate of resilience indicators, calculated over 100 repetitions.

- Discontinuous increase

We investigate a loss of resilience in a discontinuous way: (i) the bifurcation parameter is stable at c_{min}=0.5 until a threshold t_s and (ii) after the threshold t_s the bifurcation parameter increases linearly from c_{min}=0.5 to c_{max}=2.2. We investigate that function for three values of the inflection point: 40% of the total time of the simulation, 60% of the total time of the simulation and 80% of the total time of the simulation (Figure S4).
We observe an overall decrease of the performance compared to a linear loss of resilience which is, again, consistent with previous studies (Dakos et al., 2015). A discontinuous increase of *c* hampers the performance of resilience measured in bursts: we observe a decrease of performance compared to a linear loss of resilience of respectively 12%, 59% and 54% for autocorrelation and 21%, 53% and 50% for variance, for all three levels of t_s. A stepwise increase of *c* also affects the rolling window approach especially scenario III, with a decrease of performance of respectively 4%, 85% and 85% for all three levels of lambda for autocorrelation and 1%, 93% and 78% for all three levels of lambda for variance. The truncated scenario I does relatively better here as it focuses on the last part of the time series, where there is still change in this scenario.

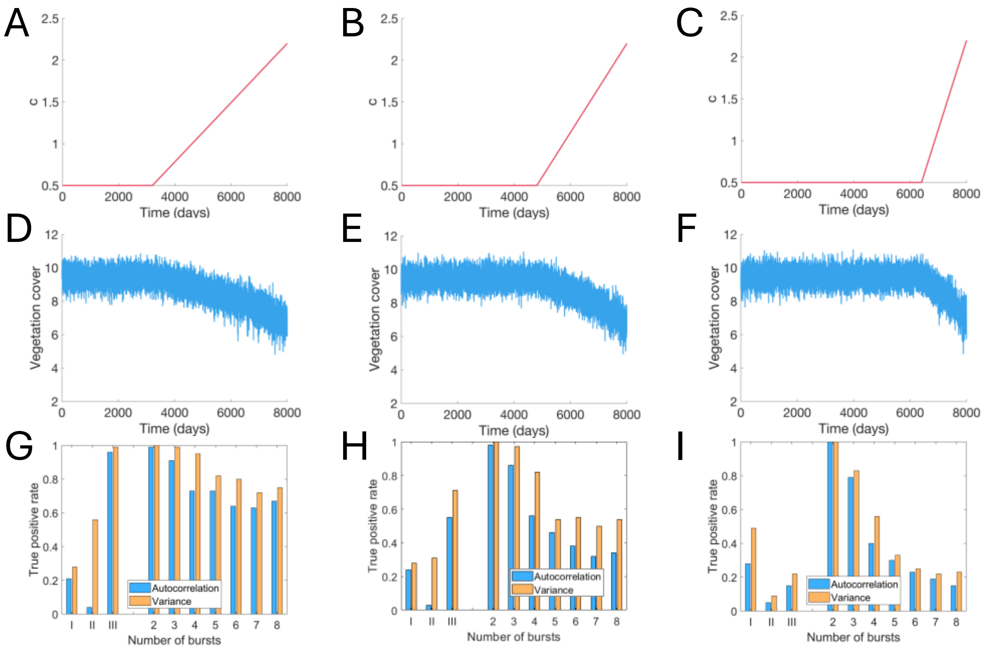

Fig. S4. Example of time series with a stepwise increase of the bifurcation parameter, and corresponding performances for the rolling window approach and the burst approach. (A) Time series of the bifurcation parameter c, stable for the first 40% of simulation time and then increasing linearly. (B) Time series of the bifurcation parameter c, stable for the first 60% of simulation time and then increasing linearly. (C) Time series of the bifurcation parameter c, stable for the first 80% of simulation time and then increasing linearly. (D) Example time series of the vegetation cover, with c increasing after 40% of the simulation time. (E) Example time series of the vegetation cover, with c increasing after 60% of the simulation time. (F) Example time series of the vegetation cover, with c increasing after 80% of the simulation time. (G) True positive rate of resilience indicators, for c increasing after 40% of the simulation time, calculated over 100 repetitions. (H) True positive rate of resilience indicators, for c increasing after 60% of the simulation time, calculated over 100 repetitions. (I) True positive rate of resilience indicators, for c increasing after 80% of the simulation time, calculated over 100 repetitions.

Autocorrelated noise

We add autocorrelated (red) noise to the model, following the equation:

$$rednoise(t) = \left(1 - \frac{1}{\lambda}\right)(rednoise(t-1) - rednoise_0) + \ rednoise_0 + \ \beta\ \sigma_E$$

with λ the period of the noise, β its extent and $rednoise_0$ its mean (here =0).
We calculate β using the formula from Ives et al. (2003), to reach a standard deviation *SD* of the noise of 0.5, comparable to the simple model with white noise: $\beta = SD\sqrt{\frac{1}{\lambda} - \frac{1}{\lambda^2}}$

Additionally, since a beta too large can lead to flickering, incompatible with the use of resilience indicators, we subtract an extra 0.05 to the calculated β (Van Der Bolt et al., 2018).
We measure the performance of the rolling window approach (scenario I, II and III) and of the burst approach for three levels of lambda: 5, 10 and 20 (Figure S5). The presence of autocorrelated noise hampers the performance of resilience measured in bursts: we observe a decrease of performance of respectively 10%, 14% and 18% for autocorrelation and 4%, 11% and 11% for variance, for all three levels of lambda. Red noise also affects the rolling window approach especially scenario I, with a decrease of performance of respectively 62%, 69% and 65% for autocorrelation and 36%, 68% and 71% for variance, for all three levels of lambda.

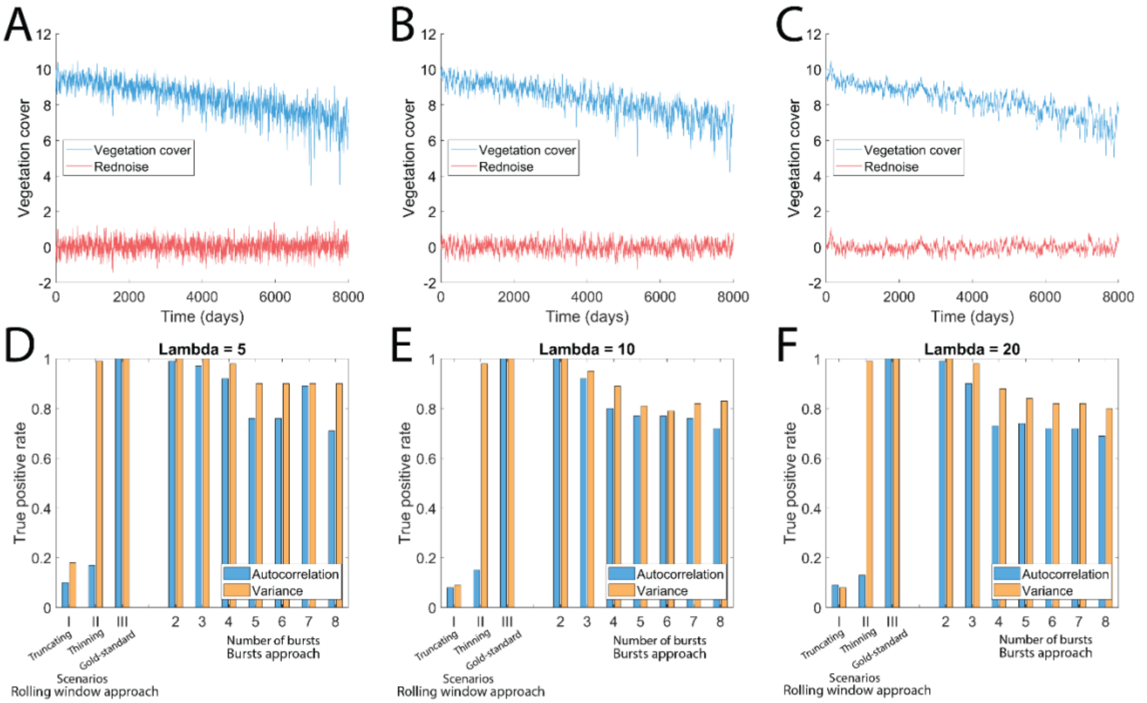

Fig. S5. Example of time series with rednoise, and corresponding performances for the rolling window approach and the burst approach. (A) Time series of the vegetation cover and the corresponding rednoise, λ=5. (B) Time series of the vegetation cover and the corresponding rednoise, λ=10. (C) Time series of the vegetation cover and the corresponding rednoise, λ=20. (D) True positive rate of the time series with rednoise, λ=5, calculated over 100 repetitions. (E) True positive rate of the time series with rednoise, λ=10, calculated over 100 repetitions. (F) True positive rate of the time series with rednoise, λ=20, calculated over 100 repetitions.

Periodic fluctuations

In this model, the grazing rate is subject to periodic forcing of amplitude *S* defined as:
$S(t) = A \times \sin(\frac{t}{p \times 2\pi})$ and $c(t) = c_{min} + (c_{max} - c_{min})\frac{t}{t_{max}} + S(t)$.
With *A* the amplitude of the periodic pattern and *p* its period. We set A=0.5 and p=3000. c_{min} and c_{max} have the same values as in the main manuscript. For both the burst and the rolling window approach, we detrend the subsampled data using a gaussian kernel, similarly to the other datasets.

The presence of periodic fluctuations hampers the performance of resilience measured in bursts compared to the model presented in the main text: we observe an overall decrease of performance of 3% for autocorrelation and 3% for variance (Fig S6). This is especially visible when the bursts are sampled without taking into account the period of the fluctuations: here, for 5 bursts, the performance of the burst approach decreases with 13% and 10% (resp. for autocorrelation and variance) compared to the dataset without periodic fluctuations (Fig S6B).

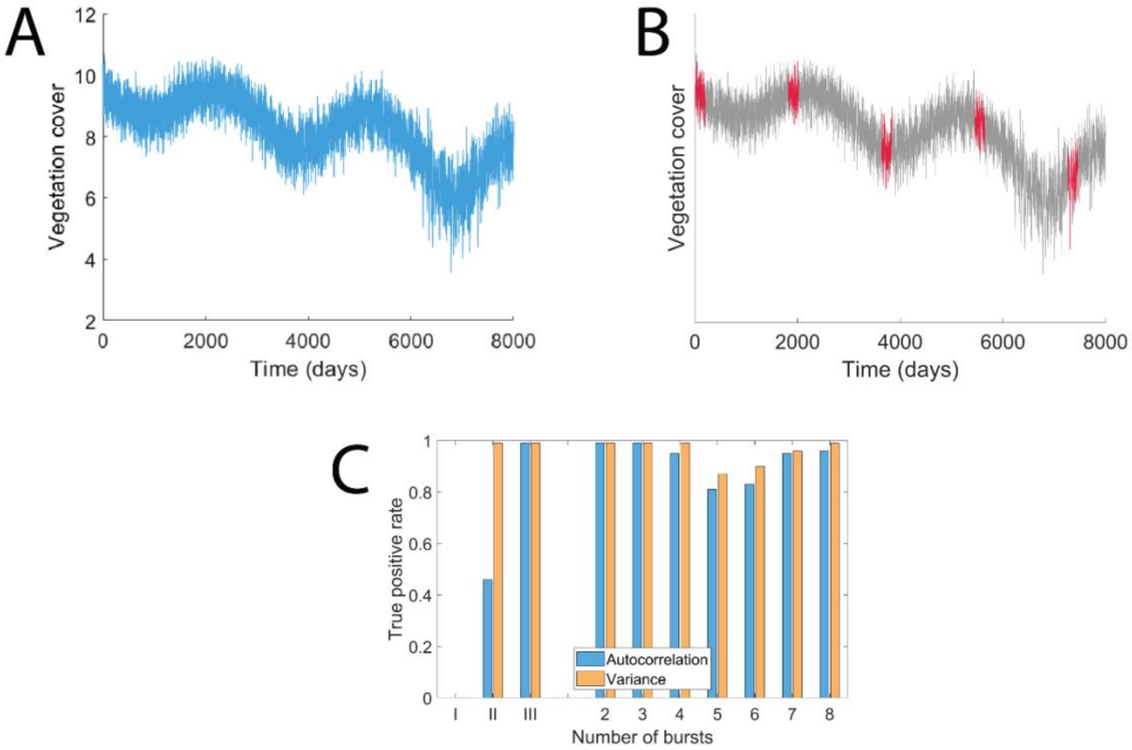

Fig. S6. Example of time series with periodic fluctuations, and corresponding performances for the rolling window approach and the burst approach. (A) Time series of vegetation cover with periodic fluctuations. (B) Example of subsampling 5 bursts, without taking into account the period of the data. (C) True positive rate of resilience indicators, calculated in data simulated using the model with periodic forcing, over 100 repetitions.

Anticipation of the collapse of the AMOC in a simulated dataset

To explore how the burst approach behaves in a more complex model, we used time series of the AMOC generated using the FAMOUS model. For more details about the model, see Hawkins et al. (2011). The model simulates the AMOC subject to a hosing experiment, causing the AMOC to collapse (Boulton et al., 2014). The time series represent the annual mean meridional overturning transport over 2383 years, and a collapse is observed at 800 years. Time series of the AMOC are generated at 49 different latitudes, between -33°N and 86.25°N. After detrending the data using a Nadaraya-Watson kernel regression, we try to detect early warning signs of the collapse using both a rolling window and a burst approach, using the different latitudes as repetitions (Fig. S7). We found that the signal in autocorrelation and especially variance is weak as we only detect the change in 34% and 8% of the repetitions (for resp. autocorrelation and variance) when using the whole dataset and a rolling window approach (scenario III, gold-standard). Surprisingly, this is slightly worse than the result of the thinning scenario (II), where the collapse is detected in 43% and 12% of the repetitions (for resp. autocorrelation and variance). The burst approach also performs poorly, with 22%, 6%, and 4% of detection for autocorrelation and 10%, 2% and 2% of detection for variance for 2, 3, and 4 bursts, respectively. Thus, when the trend in resilience indicators is weak for the rolling window approach, the burst approach does not do better.

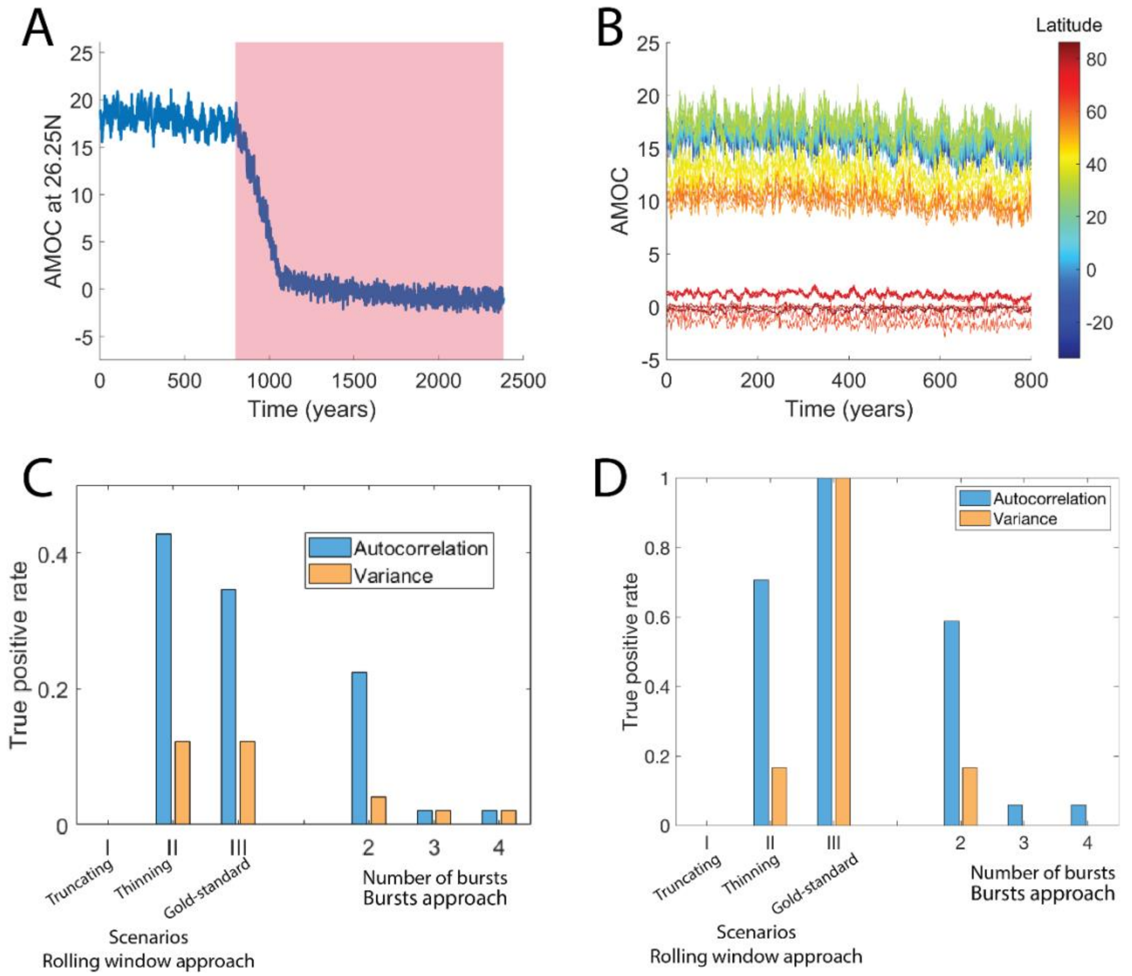

Fig. S7. Analyses of the AMOC data generated using the FAMOUS model (Boulton et al., 2014; Hawkins et al., 2011). (A) Time series of the AMOC at 26.25N over ~2300 years. The tipping point, indicated by the red shaded region, happens at 800 years. (B) Time series of the AMOC before the tipping point, for different 49 latitudes. (C) True positive rate of resilience indicators calculated in the AMOC time series. (D) True positive rate of resilience indicators calculated for the latitudes that had a significant result when using the full dataset (scenario III).

Anticipation of a depressive episode in a dataset

Trend in autocorrelation

Using the dataset described in Methods – Dataset, we tried to anticipate a depressive episode using time series of daily experiences of a patient. Autocorrelation showed a weak signal due to the inconsistent sampling interval, as the daily experiences were sampled at random times of the day and no monitoring was done at night. To overcome this issue, we tried different pre-processing of the data: detrend for daily cycles (by subtracting the mean of each day), not take into consideration in the calculations consecutive data points measured with an interval bigger than 4 hours and 6 hours, aggregate the data per day (using mean), reducing the resolution (scenario B for the rolling window approach, see methods) and use all the data. The strongest trend in autocorrelation was observed when using aggregated data per day, suggesting that indeed daily cycles hamper the use of autocorrelation for this dataset.

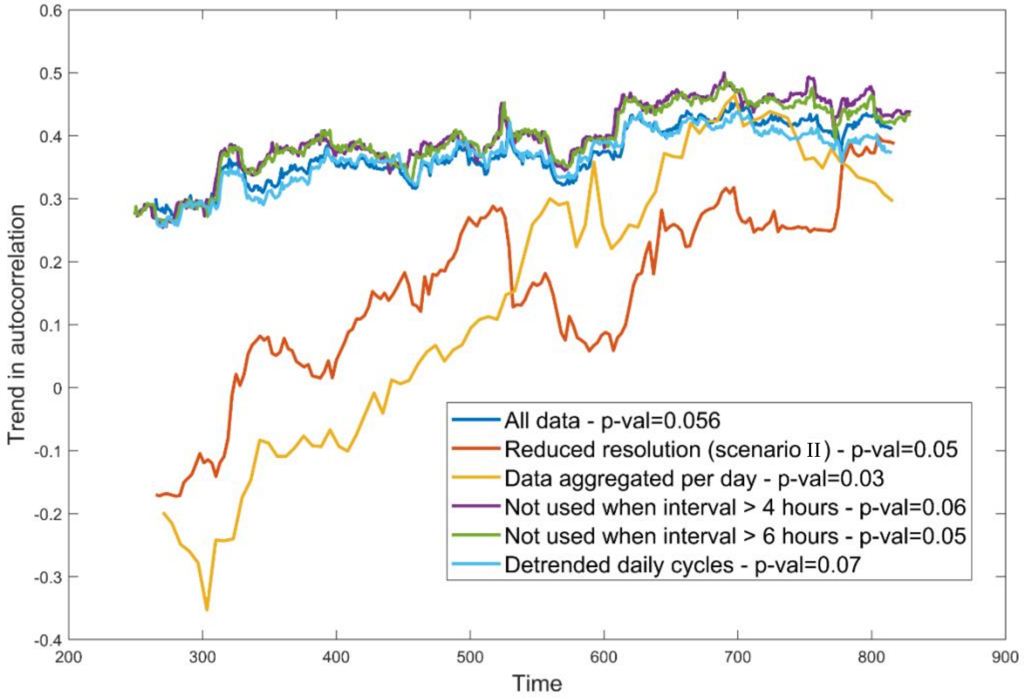

Fig. S8. Estimation of the trend in autocorrelation for different pre-processing of the data: (1) dark blue: raw data, (2) orange: scenario II of the rolling window approach (see methods), resolution is reduced by subsampling every 3rd datapoint, (3) yellow: aggregate data per day using the mean, (4) purple: consecutive data points measured with an interval bigger than 4 hours are not taken into consideration in the calculation of autocorrelation, (6) green: purple: consecutive data points measured with an interval bigger than 6 hours are not taken into consideration in the calculation of autocorrelation, (7) light blue: data are detrended for daily cycles by subtracting the mean of each hour to the corresponding data points.

Sensitivity to the size of the rolling window

We investigated the sensitivity of the trend in autocorrelation and variance to the size of the rolling window. Variance was insensitive to the size of the rolling window, but autocorrelation was. We picked a rolling window of 30% for all analyses in the empirical dataset.

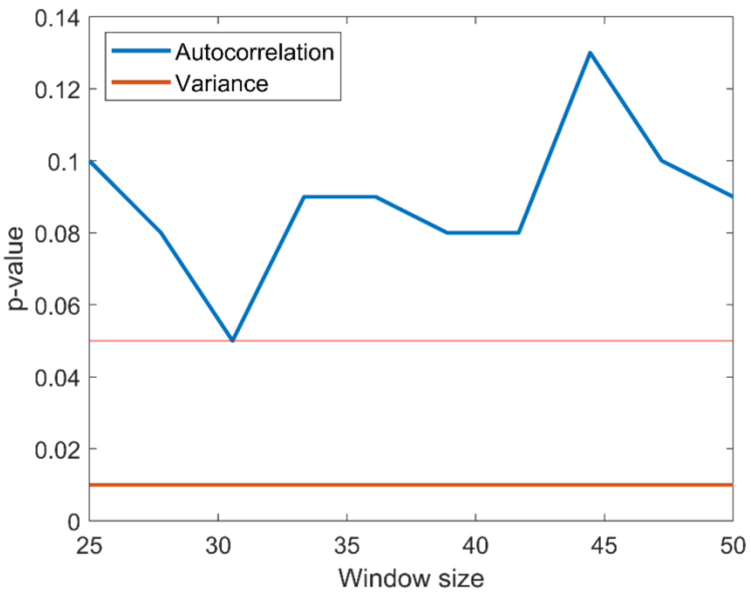

Fig S9. p-value of the trend of autocorrelation and variance for different sizes of the rolling window**,** in data projected on the first component of a PCA analysis

Scenarios

Additionally, we investigated several tangible monitoring scenarios to illustrate how bursts of measurement could have reduced the sampling intensity for the patient.

Reducing the sampling to twice two weeks of measurements would have still signaled an upcoming transition, and would have been more convenient for the patient.

Table S1. Results of different sampling scenarios to anticipate depressive episodes in the daily experiences dataset

Number of bursts	Duration of one burst	Interval between each pair of bursts	Total number of datapoints	p-value of variance
2	2 weeks	1 month	190	0.019
2	2 weeks	2 months	173	0.003
2	12 days	1 month	167	0.036
2	12 days	2 months	149	0.004
3	2 weeks	1 month	280	0.001
3	12 days	1 month	245	0.008

Effect of the total number of data points on the optimal sampling strategy when the total number of data points is fixed

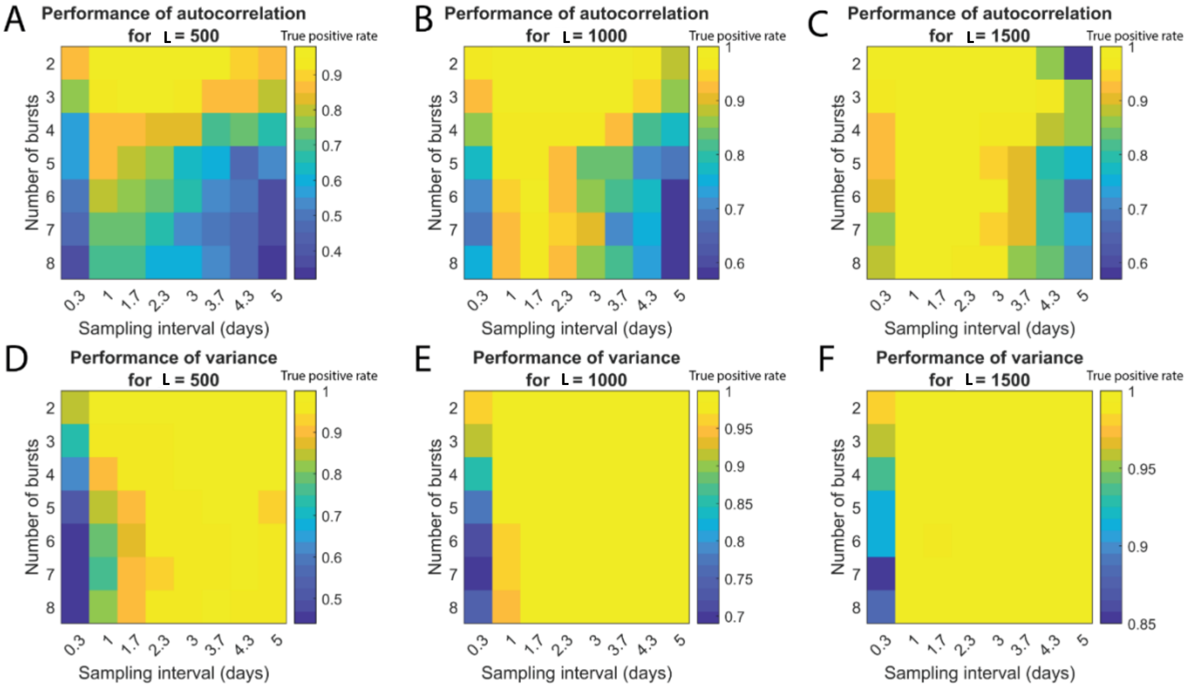

Fig S10. True positive rate of the bursts approach depending on the number of bursts n and sampling interval Δ_1, for three values of total data length L (500, 1000 and 1500). The true positive rate is estimated using the time series generated with the vegetation model, over 100 repetitions. The total number of data points was constant regardless of the number of bursts. The bursts are equally spaced in time, from the start to the end of the time series. (A) Performance of the autocorrelation for L=500. (B) Performance of the autocorrelation for L=1000. (C) Performance of the autocorrelation for L=1500. (D) Performance of the variance for L=500. (E) Performance of the variance for L=1000. (F) Performance of the variance for L=1500.

Effect of the number of data points per burst on the optimal sampling strategy when the number of data points per burst is fixed

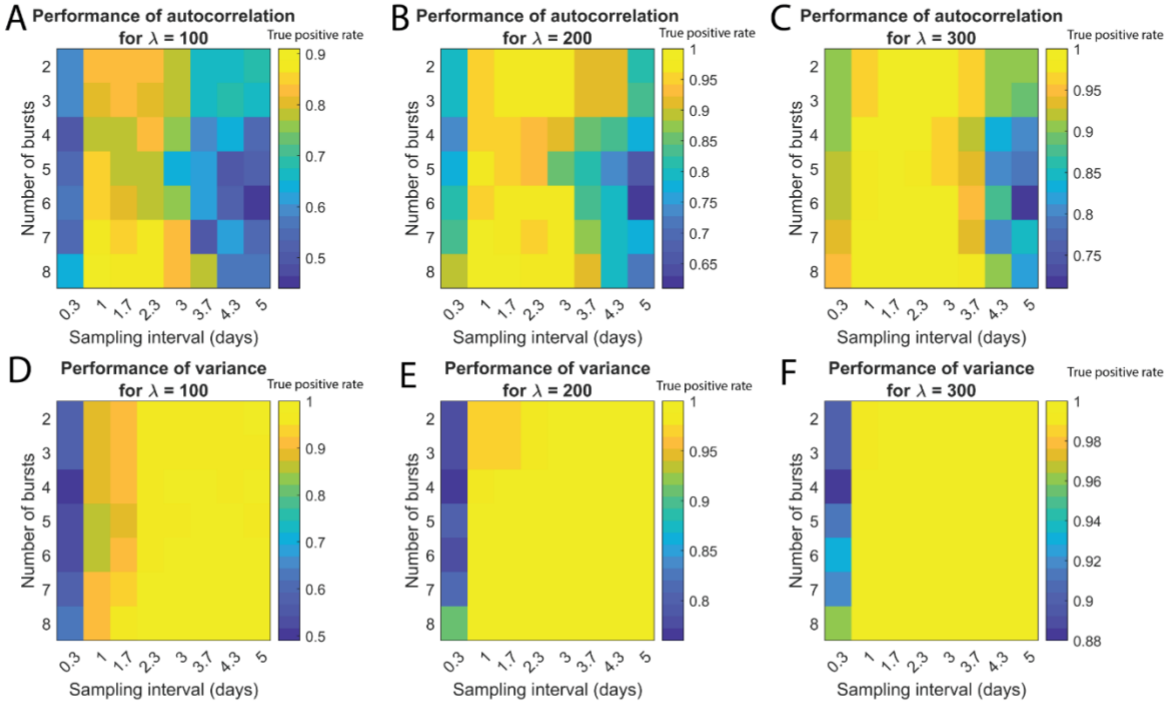

Fig S11. True positive rate of the bursts approach depending on the number of bursts n and sampling interval Δ_1, for three values of number of values per burst λ (100, 200 and 300). The true positive rate is estimated using the time series generated with the vegetation model, over 100 repetitions. The number of data points per burst was constant regardless of the number of bursts. The bursts are equally spaced in time, from the start to the end of the time series. (A) Performance of the autocorrelation for λ =100. (B) Performance of the autocorrelation for λ =200. (C) Performance of the autocorrelation for λ =300. (D) Performance of the variance for λ =100. (E) Performance of the variance for λ =200. (F) Performance of the variance for λ =300.

Lead time of prediction

Due to the distribution of bursts in the master time series, a low number of bursts showed a greater performance. This is partly explained by the fact that bursts are spread from the start to the end of the master time series. Thus, in our analyses, a lower amount of bursts results in a larger interval between the bursts. However, in real-life, one may want to detect an upcoming critical transition as early as possible. Then, every time a new burst of data is obtained, the analysis of resilience indicators will be run again with this new data. In that case, having more bursts can have the advantage of being able to anticipate the upcoming transition earlier. We estimate the lead time by measuring the minimal number of bursts within a subset still leading to the detection of a significant loss of resilience, and then measure the corresponding distance between the last data point and the critical transition. We measure the lead time over 100 repetitions and plot the average earliness over all the repetitions (figure S6 and S7). By definition, the lead time is zero for two bursts because we sample bursts from start to end of the time series, thus the second burst is placed right before the critical transition resulting in a lead time of 0 days. We observe that a higher number of bursts leads indeed to an earlier detection of the critical transition, highlighting an advantage of monitoring more bursts.

When the total number of data points L is fixed

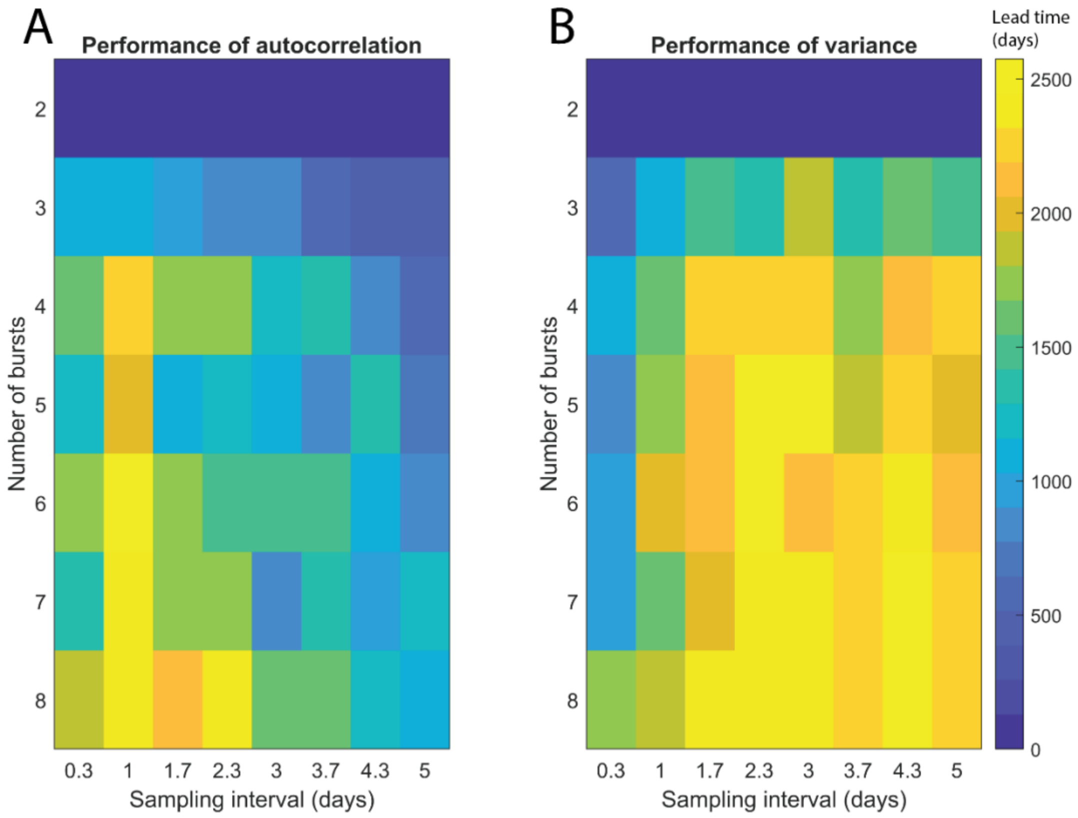

Fig S12. Lead time of prediction depending on the number of bursts n and sampling interval Δ_1. The lead time is defined as the time between the last collected data points leading to a significant detection of the loss of resilience and the critical transition. We used the time series generated with the mode for the analyses, over 100 repetitions. The total number of data points was constant (L=1000), regardless of the number of bursts. The bursts are equally spaced in time. (A) Lead time of the autocorrelation. (B) Lead time of the variance.

When the number of data points per burst λ is fixed

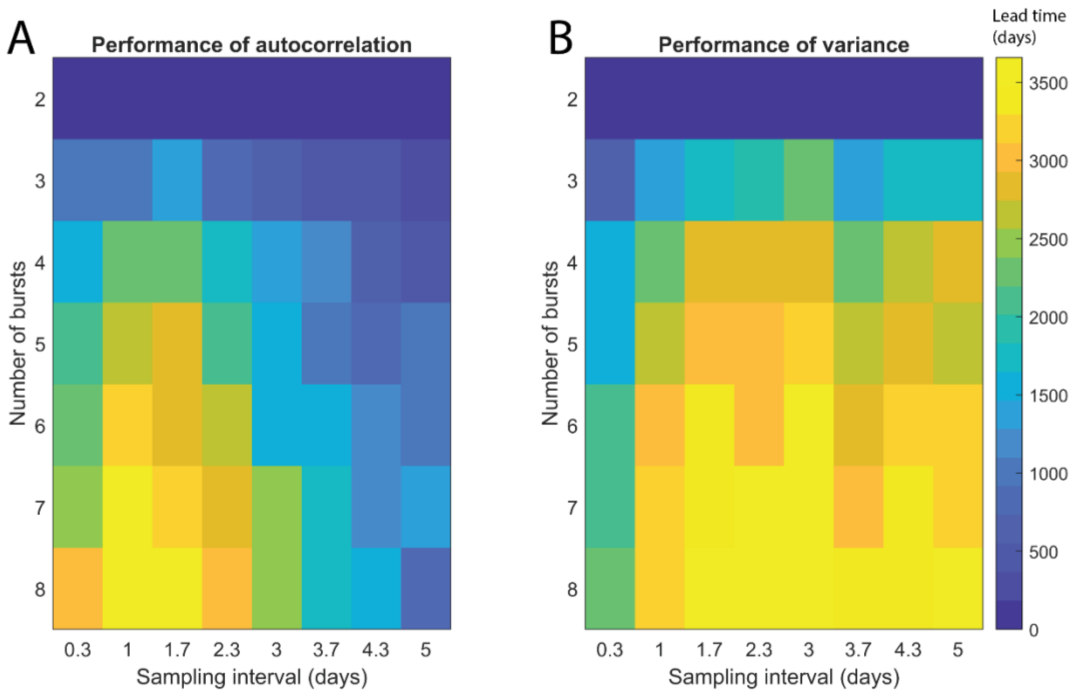

Fig S13. Lead time of prediction for autocorrelation and variance depending on the number of bursts n and sampling interval Δ_1. The lead time is defined as the time between the last collected data points leading to a significant detection of the loss of resilience and the critical transition. We used the time series generated with the mode for the analyses, over 100 repetitions. The number of data points per burst was constant (λ=200), regardless of the number of bursts. The bursts are equally spaced in time. (A) Lead time of the autocorrelation. (B) Lead time of the variance.
